# Acute and Long-Term Treatment With Dapagliflozin and Association With Serum Soluble Urokinase Plasminogen Activator Receptor

**DOI:** 10.3389/fphar.2022.799915

**Published:** 2022-04-27

**Authors:** Viktor Rotbain Curovic, Morten B. Houlind, Tine W. Hansen, Jesper Eugen-Olsen, Jens Christian Laursen, Mie K. Eickhoff, Frederik Persson, Peter Rossing

**Affiliations:** ^1^ Steno Diabetes Center Copenhagen, Herlev, Denmark; ^2^ Department of Clinical Research, Hvidovre Hospital, Hvidovre, Denmark; ^3^ Department of Clinical Medicine, University of Copenhagen, Copenhagen, Denmark

**Keywords:** type 1 diabetes, inflammation, suPAR, soluble urokinase receptor, clinical trial, randomized controlled trial, type 2 diabetes, biomarker

## Abstract

**Background:** Elevated soluble urokinase plasminogen activator receptor (suPAR) is highly associated with increased risk of diabetic complications. Dapagliflozin is a drug inhibiting the sodium-glucose co-transporter 2 in the kidney to decrease blood glucose, while also decreasing risk of kidney disease, heart failure, and death. Therefore, we have investigated suPAR as a monitor for treatment effect with dapagliflozin in diabetes.

**Methods:** suPAR was measured in two double-blinded randomized clinical cross-over trials. The first trial investigated the effect of a single dose dapagliflozin 50 mg or placebo 12 h after intake, in individuals with type 1 diabetes and albuminuria. The second trial investigated the effect of a daily dose dapagliflozin 10 mg or placebo for 12 weeks, in individuals with type 2 diabetes and albuminuria. suPAR was measured in serum samples taken, in the acute trial, after treatment with dapagliflozin and placebo, and in the long-term trial, before and after treatment with dapagliflozin and placebo. Effect of dapagliflozin on suPAR levels were assessed using paired *t*-test.

**Results:** 15 participants completed the acute trial and 35 completed the long-term trial. Mean difference in suPAR between dapagliflozin and placebo in the acute trial after 12 h was 0.70 ng/ml (95% CI: 0.66; 1.33, *p* = 0.49). In the long-term trial the mean difference was 0.06 ng/ml (95% CI -0.15; 0.27, *p* = 0.57).

**Conclusion:** Based on our findings we conclude that suPAR is not a feasible marker to monitor the effect of treatment with dapagliflozin. Thus, a further search of suitable markers must continue.

## Introduction

Soluble urokinase plasminogen activator receptor (suPAR) levels reflect inflammation and immune activation (Thunø et al., 2009). Elevated suPAR levels are robustly associated with increased morbidity and mortality across acute and chronic diseases (Hayek et al., 2015; Rasmussen et al., 2016; Hayek et al., 2020; Hodges et al., 2020). Moreover, elevated suPAR levels are associated with an increased risk of diabetic cardiovascular and kidney complications across long-term follow-up (Rasmussen et al., 2016; RotbainCurovic et al., 2019). Sodium glucose co-transporter receptor inhibitors (SGLT2-I), such as dapagliflozin, are on the path to revolutionize treatment of diabetic nephropathy, chronic kidney disease and heart failure (Neal et al., 2017; McMurray et al., 2019; Heerspink et al., 2020). However, the mechanisms of SGLT2-I’s reno-protective effects are largely unknown, and we have no feasible way to predict or monitor the treatment effect in the clinical setting. While glucose and albuminuria levels are assessed regularly during standard diabetes care and decrease during SGLT2-I treatment, neither marker can fully explain the effect on renal endpoints. Theories concerning the reno-protective effect of SGLT2-I treatment range from, amongst others, hemodynamic alterations (Cherney et al., 2014), lowering of sympathetic nervous system overactivity (Sano, 2018), and possible amelioration of renal hypoxia (Laursen et al., 2021); the latter suggested by reduction of inflammation and fibrosis (Packer, 2021). Likewise, studies have shown the presence of SGLT2 receptors on endothelial plaques (D'Onofrio et al., 2021), as well as indicating an added protective benefit on inflammatory burden and clinical outcomes after coronary artery bypass grafting across 5 years (Sardu et al., 2021). Given the potent, unspecific, risk associations between suPAR and diabetic complications, we consider suPAR to be a fitting candidate to investigate during treatment with dapagliflozin, which is markedly effective in reducing multiple diabetic complications. We hypothesized that suPAR can be used to monitor the effect of treatment with dapagliflozin as an overall treatment effect marker due to its unspecific risk associative properties.

In this post-hoc study of two randomized controlled double-blinded cross-over trials, we have measured the effect of dapagliflozin treatment in an acute and a long-term setting, respectively, on circulating suPAR in individuals with diabetes and albuminuria.

## Methods

### Study Design—Acute Trial

In this manuscript, we have included participants from two randomized controlled trials. The first trial was a randomized controlled double-blinded cross-over trial investigating the acute effect (12 h) of a single 50 mg dose of dapagliflozin or placebo on renal oxygenation in 15 individuals with type 1 diabetes, separated by a 2-week washout period (NCT04193566). The trial was conducted between 3 February 2020 and 23 October 2020 in Copenhagen, Denmark. The study design have been published previously (Laursen et al., 2021), and a complete list of inclusion and exclusion criteria can be found in Supplementary Table S1. In summary, participants were considered eligible if they had type 1 diabetes and albuminuria defined as a urine albumin creatinine ratio (UACR) ≥30 mg/g in two out of three consecutive first morning void urine samples prior to randomization. Participants were randomly assigned in a 1:1 ratio to dapagliflozin-placebo or placebo-dapagliflozin treatment order. Participants received a single dose of dapagliflozin 50 mg or placebo 12 h before measuring suPAR in a serum sample. Thereafter, they proceeded to a 2-weeks washout period, before cross-over and receiving dapagliflozin 50 mg or placebo, depending on randomization sequence.

### Study Design—Long-Term Trial

The second trial was a randomized controlled double-blinded cross-over trial originally designed to investigate the effect of 10 mg dapagliflozin on the urinary proteomics classifier CKD273 (NCT02914691) in 36 individuals with type 2 diabetes and albuminuria, across 12 weeks of treatment. The trial was conducted between 1 August 2015 and 5 July 2017 in Copenhagen, Denmark. The study design has been described before (Eickhoff et al., 2020) and a complete list of inclusion and exclusion criteria can be found in Supplementary Table S2. In short, participants were eligible if they had type 2 diabetes, an estimated glomerular filtration rate (eGFR) ≥45 ml/min/1.73^2^, HbA1c > 58 mmol/mol (7.5%), and UACR ≥30 mg/g in two out of three consecutive first morning void urine samples. Participants were excluded if they had a cardiovascular event 2 months prior to randomization, or if they had congestive heart failure (NYHA IV or unstable or acute congestive heart failure).

### Ethics Statement

Participants from both trials were recruited from the outpatient clinic at Steno Diabetes Center Copenhagen, Denmark. All participants gave written informed consent. The trials were conducted according to Good Clinical Research Practice and the Helsinki declaration, and were approved by the regional ethics committee of The Capital Region, Denmark (acute trial: H-19052662; long-term trial: H-15006370).

### Blinding and Randomization

In the acute trial blinding was performed using an allocation schedule with one block of 20 sequences generated from the webpage www.randomization.com. The allocation schedule, sealed envelopes with unblinding-details, and 20 sequentially numbered sealed opaque plastic bags containing study medication were produced by Glostrup Pharmacy, Copenhagen, Denmark. In the long-term trial, blinding and randomization of the study medication was carried out by The Capital Region Pharmacy, Copenhagen, Denmark, including storage of sealed envelopes with unblinding-details and packaging of study medication. During both trials, study participants and investigators were masked to the study medication with unblinding taking place only after all study procedures had been finalized.

### Outcomes

The present study is a post-hoc analysis of the effect of dapagliflozin treatment on serum suPAR levels in two randomized clinical trials. The outcome is difference in suPAR level after treatment with dapagliflozin and placebo; after 12 h in the acute trial, and after 12 weeks in the long-term trial. Due to this being an explorative post-hoc analysis sample size calculation was performed.

In the acute trial, the primary outcome was change in renal oxygenation measured with quantitative MRI on a 3-T Philips Achieva scanner (Liss et al., 2013). Secondary outcomes included change in blood pressure, blood oxygen saturation, heart rate, autonomic nerve function assessed by baroreflex sensitivity, mitochondrial oxygen consumption rate, and inflammatory biomarkers. In the long-term trial, the primary outcome was change in the urinary proteomic classifier CKD273. Urine proteomic quantification was performed by capillary electrophoresis–mass spectrometry analysis and a predefined renal risk profile based on 273 peptides (CKD273) was calculated as previously described (Tofte et al., 2020; RotbainCurovic et al., 2021). Secondary outcomes included change in echocardiographic measures, cardiac-specific biomarkers, and 24-h blood pressure.

### Clinical Measurements

suPAR (suPARnostic ELISA, ViroGates, Birkerød, Denmark) was measured after each treatment period (dapagliflozin and placebo) in the acute trial and at the beginning and end of each treatment period in the long-term trial. In the acute trial, interleukin 6 (IL6) and tumor necrosis factor alpha (TNF-a) was measured in plasma with the Olink^®^ Target 96 Inflammation panel (Olink, Uppsala, Sweden) (Assarsson et al., 2014). Biomarker levels are shown as the Normalized Protein Expression (NPX) which is a relative arbitrary unit on a log2 scale. In the long-term trial, IL6 and TNF-a was measured in plasma samples using ELISA (Quantikine HS, R&D Systems, Abingdon, Oxon, United Kingdom) and are presented quantitatively as pg/ml. Measurements were performed in samples collected at end-of-treatment for each treatment period in the acute trial, and at baseline and end-of-treatment for each treatment period in the long-term trial.

### Statistical Analysis

Baseline characteristic are presented as means (standard deviations (SD)) for normally distributed variables. Non-normal distribution is presented as median (inter-quartile range). The distribution of suPAR was checked in both trials and deemed normally distributed; thus, dapagliflozin treatment effect on suPAR was assessed using paired *t*-test for both trials, and presented with mean (95% confidence interval (CI)) difference between suPAR level after dapagliflozin and after placebo treatment. Furthermore, paired t-tests were performed assessing the change in suPAR level after dapagliflozin treatment, and after placebo treatment, respectively; only in the long-term trial as baseline analysis of suPAR level were not available in the acute trial. Sensitivity analyses including adjustment for eGFR, HbA1c, and systolic blood pressure, individually and combined, were performed by linear regression and sensitivity analyses investigating potential carry over effect were performed using mixed effects models fitted using treatment, sequence, and period as fixed effects and random intercepts for all participants. Furthermore, correlation matrices presented with Pearson’s R were calculated for suPAR, IL6, TNF-a, and the respective primary outcome measures for the acute and long-term trials. All statistical analyses were performed using R version 4.1.0 (R Core Team, Vienna, Austria) and RStudio version 1.4.1106 (RStudio Team, Boston, MA, United States).

## Results

There were 15 participants who completed the acute trial, and 35 who completed the long-term trial. There were more men (67 and 89% in the acute and long-term trial, respectively) than women in both trials and all participants were Caucasian apart from two participants in the long-term trial. Mean diabetes duration differed radically, due to the acute trial participants having type 1 diabetes, and the long-term type 2 diabetes (39 ± 16 years vs. 16 ± 5 years), as did insulin use, for the same reason (100 vs. 66%). Mean eGFR was lower in the acute trial (73 ± 32 ml/min/1.73 m^2^) compared to the long-term trial (84 ± 19 ml/min/1.73 m^2^), however, the relationship was inverse for UACR, where the participants in the long-term trial three times higher median (154 (94; 329) mg/g) than in the acute trial (43 (21; 58) mg/g) (Table 1). The prescription of renin-angiotensin-aldosterone system inhibitors (87 vs. 100%) and diuretics (60 vs. 61%) were comparable in the trials.

**TABLE 1:** Baseline characteristics of participants in the acute and long-term trials. Dichotomous variables are presented as n (%), continuous as mean ± SD, and non-normal continuous variables as median (inter-quartile range). SuPAR values were not measured at baseline in the acute trial. HbA1c: Glycated hemoglobin; eGFR: estimated glomerular filtration rate; BP: blood pressure; UACR: urinary albumin creatinine rate; suPAR: soluble urokinase plasminogen activator receptor; RAASi: renin-angiotensine-aldosterone system inhibitor.

	Acute Trial (n = 15)	Long-Term Trial (n = 35)
Female, n (%)	33 (5)	11 (4)
Age, years	58 ± 14	64 ± 8
Race, n (%)	-	-
***Caucasian***	15 (100)	33 (94)
***Other***	0 (0)	2 (6)
Diabetes duration, years	39 ± 16	16 ± 5
suPAR ng/ml	-	3.44 (2.49; 4.35)
Creatinine, µmol/l	105 ± 55	82 ± 23
eGFR, ml/min/1.73m^2^	73 ± 32	84 (19)
UACR, mg/g	43 (21; 58)	154 (94; 329)
Systolic BP	138 (16)	141 (16)
HbA1c, mmol/mol	61 ± 7	73 ± 15
HbA1c, %	7.7 ± 0.6	8.9 ± 1.4
RAASi, n (%)	13 (87)	35 (100)
Diuretics, n (%)	9 (60)	18 (51)
Insulin, n (%)	15 (100)	23 (66)

**FIGURE 1:**
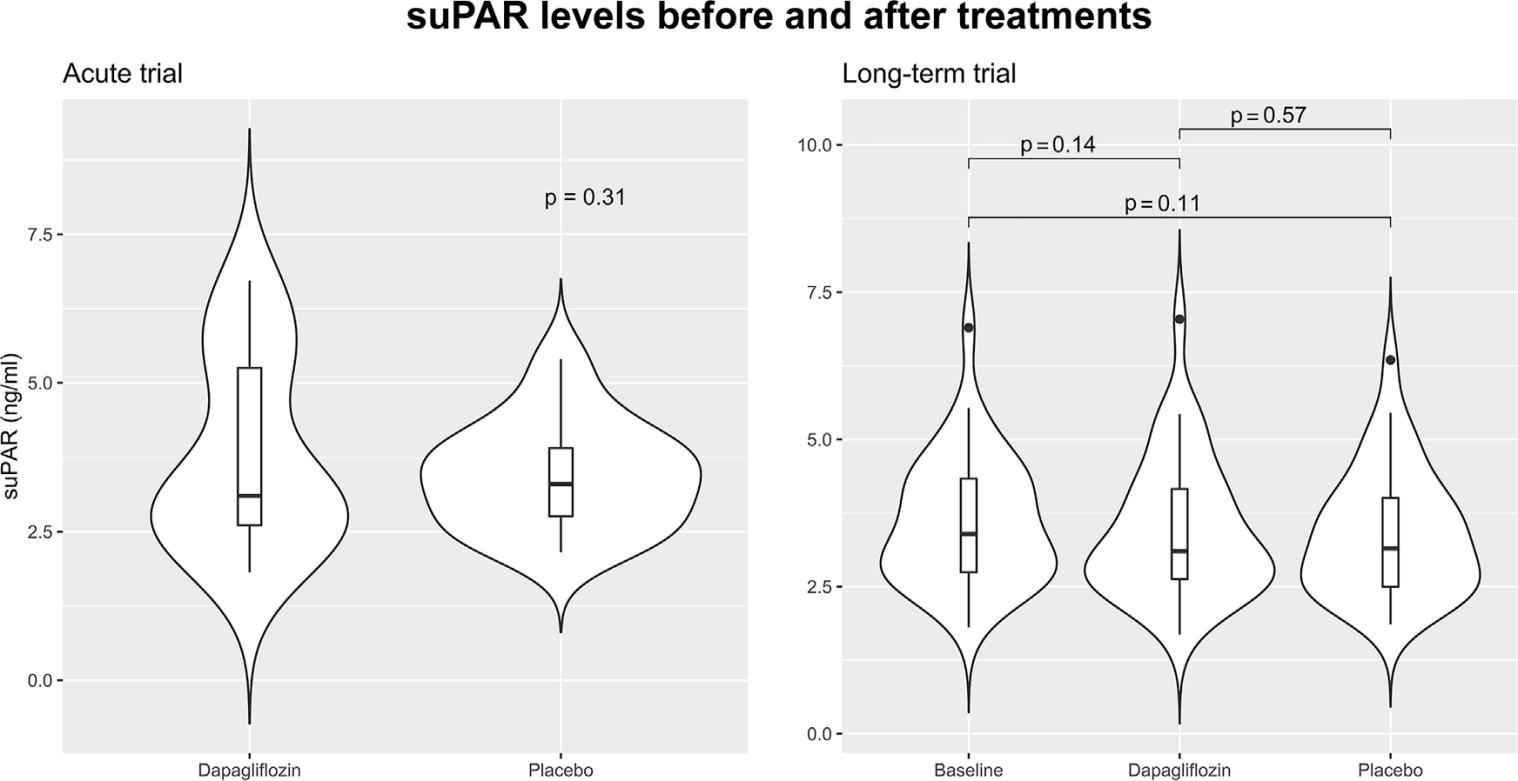
Violin plots visualizing the distribution of soluble urokinase plasminogen activator receptor (suPAR) in the acute and long-term trials. In the acute trial suPAR levels 12 h after treatment with a single dose of 50 mg dapagliflozin are shown. In the long-term trial suPAR level is shown at baseline, after 12 weeks treatment with dapagliflozin 10 mg daily, and after 12 weeks treatment with placebo. Baseline data of suPAR level was not available in the acute trial. All comparisons are performed using paired t-tests.

In the acute trial, the mean ± standard deviation of suPAR was 3.78 ± 1.62 ng/ml and 3.44 ± 0.91 ng/ml, 12 h after dapagliflozin or placebo administration. Mean difference in suPAR after 12 h was 0.70 ng/ml (95% CI: 0.66; 1.33, *p* = 0.49). Change in suPAR in the long-term trial after 12 weeks of treatment with dapagliflozin was −0.13 ng/ml (95% CI -0.31; 0.05, *p* = 0.14), and placebo −0.19 ng/ml (−0.42; 0.04, *p* = 0.11), mean difference was 0.06 ng/ml (95% CI −0.15; 0.27, *p* = 0.57) (Figure 1).

As suPAR level can be affected by other variables such as kidney function, we did sensitivity analyses assessing change in suPAR level after treatment with dapagliflozin or placebo, with linear regression models adjusting for eGFR, HbA_1c_, systolic blood pressure, and UACR, individually and combined in each of the trials, and using mixed effects models to test for carry over effect. All sensitivity analyses confirmed the results of the primary analyses.

**FIGURE 2:**
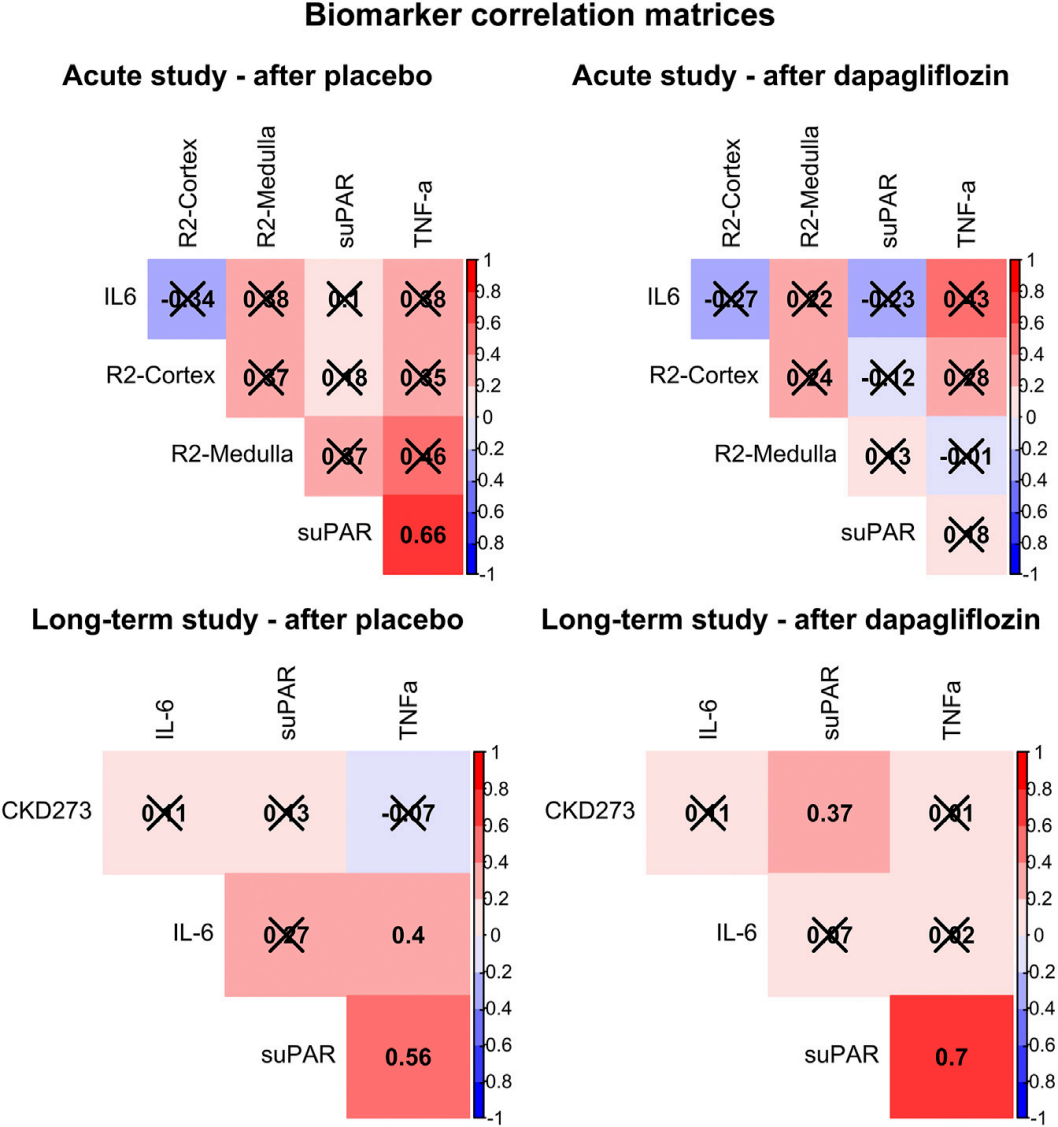
Correlation matrices showing Pearson’s R for all associations between soluble urokinase plasminogen activator receptor (suPAR), interleukin 6 (IL6), tumor necrosis factor alpha (TNF-a), and the primary outcomes for each trial (cortical renal oxygenation (R2-Cortex) and medullary renal oxygenation (R2-Medulla), and the CKD273 classifier, respectively), after treatment with placebo and dapagliflozin. Non-significant correlations are crossed over. Positive correlations are colored red, and negative colored blue.

Additionally, the relationship between levels of suPAR, IL6 and TNF-a, and the respective primary outcome in the respective trial, after dapagliflozin and placebo treatment, is presented in Figure 2. In the acute trial, the only significant correlation was found between higher suPAR and higher TNF-a, after placebo (R = 0.66, *p* = 0.015), but not after dapagliflozin treatment. In the long-term trial, this positive correlation was significant both after placebo (R = 0.56, *p* = 0.001) and after dapagliflozin (R = 0.70, *p* < 0.001) treatment. Furthermore, a significant positive correlation was shown between suPAR and CKD273 after dapagliflozin (R = 0.37, *p* < 0.050) treatment.

## Discussion

We have investigated the effect of dapagliflozin on suPAR, a sensitive inflammatory biomarker highly associated with the development of diabetic complications. Our results did not show any change in suPAR mediated by dapagliflozin treatment in neither acute nor long-term settings. As dapagliflozin exhibits a large reduction of kidney and heart disease risk (Neal et al., 2017; McMurray et al., 2019; Heerspink et al., 2020), we must conclude that its mediation operates by different mechanisms than those affecting suPAR levels. Therefore, suPAR is not suitable for the monitoring of dapagliflozin treatment effect. In addition, we showed that suPAR was generally correlated with TNF-a levels, but not with IL6, CKD273 or renal oxygenation, indicating an independence from other inflammatory and non-inflammatory risk markers.

SuPAR is an unspecific inflammatory marker and its membrane-bound form is present on a myriad of inflammatory cells (Thunø et al., 2009). Higher levels reflect a state of inflammation, which is also identified in diabetes in general (Tsalamandris et al., 2019). Although reports concerning possible anti-inflammatory effects of SGLT2-I exist (Bonnet and Scheen, 2018), results are inconsistent as well as possibly mediated by other factors relating to the effect of SGLT2-I. Atherosclerotic plaques from individuals with diabetes have been shown to be highly expressed with SGLT2 and have a higher degree of inflammation than individuals without diabetes. However, the levels of inflammation was lower for individuals with diabetes who were treated with SGLT2-I compared to those without SGLT2-I therapy (D'Onofrio et al., 2021). Likewise, it has been demonstrated that treatment with canagliflozin, another SGLT2-I, across 52 weeks significantly decreased IL6, and increased TNF-a, compared to treatment with glimepiride (a sulfonylurea) (Garvey et al., 2018), and was shown to modulate an added protective effect on both inflammatory burden and overall survival after coronary artery bypass grafting (Sardu et al., 2021); however these results are not uniformly demonstrated in other trials (Matsumura et al., 2017), including the two trials composing this study (Eickhoff et al., 2020; Laursen et al., 2021). In the present study we do not show a change in suPAR after treatment with dapagliflozin. However, we would argue that due to suPAR’s sensitivity to reflecting inflammation in a multitude of disease states, showing no change during treatment with a highly reno- and cardio-protective medication such as dapagliflozin, is an important step in revealing the mechanisms behind SGLT2-Is’ marked efficacy.

We would consider the choice to include populations with both type 1 and type 2 diabetes as a strength in our study diversifying the investigated participants and increasing generalizability, however the possibility that dapagliflozin affects suPAR levels differently across diabetes types cannot be excluded. Despite that dapagliflozin, or SGLT2-Is in general, is not yet guideline recommended treatment for type 1 diabetes, mainly due to reports of increased ketoacidosis, it will very possibly be applicable to at least some subgroups of type 1 diabetes individuals in the future (Taylor et al., 2019), constituting a need for more trials on SGLT2-Is in type 1 diabetes. . Additionally, as our long-term study only stretches across 12 weeks, there is a possibility that an even longer treatment period might affect suPAR levels, as shown for TNF-a and IL6 by Garvey et al. (Garvey et al., 2018). Likewise, we have only investigated dapagliflozin’s effect on suPAR in two small trials; larger trials with more power might find differing results. The population included in this study was moreover at a progressed stage of diabetes with higher albuminuria and lower kidney function which could affect results. However, sensitivity analyses with adjustment for albuminuria and kidney function were confirmatory. Furthermore, in the long-term trial the numerical change of suPAR after dapagliflozin treatment spanned a 95% CI: 0.15; 0.27. Comparing to our previous study investigating the baseline risk of suPAR in type 1 diabetes (median (inter-quartile range): 3.4 (2.7–4.5) ng/ml), in which we found very small separation between the lower three quartiles of suPAR in association to decline in eGFR (RotbainCurovic et al., 2019), this indicates that a larger change in suPAR would be needed for a significant risk reduction of kidney disease development or progression. Lastly, complete information regarding the medical therapy of the included participants in both studies was not available. Thus, we cannot rule out a confounding effect of concomitant medication on suPAR level. Treatment with irbesartan have been shown to lower urinary suPAR (Persson et al., 2016), but no medical treatment has previously shown change in circulating suPAR and therefore we must assume this has not impacted our results.

Based on our findings we conclude, that suPAR is not a feasible marker to monitor the effect of treatment with dapagliflozin and thus a further search of suitable markers must continue.

## Data Availability

The datasets presented in this article are not readily available because of privacy and ethical concerns according to Danish legislation. Requests to access the datasets should be directed to viktor.rotbain.curovic@regionh.dk.
