# Supplementary material for: Acute and Long-Term Treatment With Dapagliflozin and Association With Serum Soluble Urokinase Plasminogen Activator Receptor
Source: Front Pharmacol. 2022 Apr 27;13:799915. doi: 10.3389/fphar.2022.799915 (PMC9091812; doi:10.3389/fphar.2022.799915)
Supplement: Supplementary file 1 [file DataSheet1.DOCX]

## **Table 1: Full inclusion and exclusion criteria for the acute trial.**

## Inclusion criteria:

- Written informed consent must be provided before participation
- Male or female patients >18 years of age with a diagnosis of type 1 diabetes (WHO criteria)
- Urinary albumin creatinine ratio (UACR) ≥30 mg/g in 2 out of 3 consecutive samples (albuminuria) prior to randomization assessed from electronic laboratory database.
- Capable of lying in a MR-scanner for two hours

## Exclusion criteria:

- Non-diabetic kidney disease as indicated by medical history and/or laboratory findings
- Renal failure (eGFR<15 ml/min/1.73m2), dialysis or kidney transplantation
- Treatment with beta-blocking medication
- Uncontrolled arrhythmia, 2. or 3. degree AV-block or sick sinus syndrome - assessed from a standard 12-lead electrocardiogram
- Pregnancy or breastfeeding (urine HCG is performed on all fertile women)
- Systolic blood pressure < 90 or > 200 mmHg
- Patients who, in the judgement of the investigator, is incapable of participating
- Exclusion criteria for MRI
  - Claustrophobia
  - Known heart disease
  - Known lung disease
  - Have had surgery the past six weeks
  - Have foreign bodies of metal in the body (e.g. pacemaker, metal plates, metal screws)
- Exclusion criteria for arterial blood gas sampling
  - Absent pulse
  - Raynauds syndrome
  - Buergers Disease (thromboangiitis obliterans)
  - Inadequate or interrupted circulation
  - Anticoagulation treatment
  - Coagulopathies (hypo or hyper coagulable states)
  - Arterial atherosclerosis
  - Insufficient collateral perfusion
  - Partial or full thickness burns over the cannulation site
  - Synthetic arterial or vascular grafts or infection at the proposed site of cannulation

**Supplementary Table 2: Full inclusion and exclusion criteria for the long-term trial.**

Inclusion criteria:

- Male or female patients >18 years of age with a diagnosis of type 2 diabetes (WHO criteria).
- Patients must be on current stable antiglycaemic treatment with oral drugs (OAD) or insulin 4 weeks before start of study drug and throughout study duration.
- Patients must be on stable antihypertensive treatment (must include renin-angiotensin system blocking treatment) 4 weeks before start of study drug and throughout study duration.
- HbA1c >7.5 %
- Urinary albumin creatinine ratio (UACR) > 30 mg/g (in ≥2 out 3 morning spot urine collections prior to randomisation).
- eGFR ≥ 45 ml/min/1.73 m2
- Stable RAAS-blocking treatment (more than or equal to 4 weeks prior to visit 0) If not stable at visit 0, screening phase can be prolonged to 4 weeks.

Exclusion criteria:

- Current treatment with loop diuretics
- Current treatment with thiazolidinediones
- Current treatment with dapagliflozin or other SGLT2 inhibitor
- Ongoing cancer treatment
- Patients on hypertension treatment who are is not on stable antihypertensive treatment (must include renin-angiotensin system blocking treatment) 4 weeks before start of study drug and throughout study duration
- Severe hepatic insufficiency and/or significant abnormal liver function defined as aspartate aminotransferase (AST) >3x upper limit of normal (ULN) and/or alanine aminotransferase (ALT) >3x ULN
- Total bilirubin >2.0 mg/dL (34.2 μmol/L)
- Positive serologic evidence of current infectious liver disease including, Hepatitis B surface antigen and antibody and Hepatitis C virus antibody
- eGFR: <45 mL/min (calculated by MDRD formula)
- History of unstable or rapidly progressing renal disease
- Volume depleted patients. Patients at risk for volume depletion due to co-existing conditions or concomitant medications, such as loop diuretics should have careful monitoring of their volume status
- Recent Cardiovascular Events in a patient:

1. Acute Coronary Syndrome (ACS) within 2 months prior to enrolment

2.Hospitalization for unstable angina or acute myocardial infarction within 2 months prior to enrolment

3. Acute Stroke or TIA within two months prior to enrolment

4. Less than two months post coronary artery revascularization

- Congestive heart failure defined as New York Heart Association (NYHA) class IV, unstable or acute congestive heart failure. Note: eligible patients with congestive heart failure, especially those who are on diuretic therapy, should have careful monitoring of their volume status throughout the study.
- Pregnant or breastfeeding patients
- Patients who, in the judgement of the investigator, may be at risk for dehydration
